# Revisiting the expression signature of *pks15/1* unveils regulatory patterns controlling phenolphtiocerol and phenolglycolipid production in pathogenic mycobacteria

**DOI:** 10.1371/journal.pone.0229700

**Published:** 2020-05-07

**Authors:** Beatriz Ramos, Stephen V. Gordon, Mónica V. Cunha

**Affiliations:** 1 National Institute for Agrarian and Veterinary Research (INIAV, IP), Oeiras, Portugal; 2 Centre for Ecology, Evolution and Environmental Changes (cE3c), Faculdade de Ciências da Universidade de Lisboa, Lisboa, Portugal; 3 School of Veterinary Medicine and Conway Institute, University College Dublin, Dublin, Ireland; 4 Biosystems & Integrative Sciences Institute (BioISI), Faculdade de Ciências da Universidade de Lisboa, Lisboa, Portugal; Centre National de la Recherche Scientifique, FRANCE

## Abstract

One of the most important and exclusive characteristics of mycobacteria is their cell wall. Amongst its constituent components are two related families of glycosylated lipids, diphthioceranates and phthiocerol dimycocerosate (PDIM) and its variant phenolic glycolipids (PGL). PGL have been associated with cell wall impermeability, phagocytosis, defence against nitrosative and oxidative stress and, intriguingly, biofilm formation. In bacteria from the *Mycobacterium tuberculosis* complex (MTBC), the biosynthetic pathway of the phenolphthiocerol moiety of PGL depends upon the expression of several genes encoding type I polyketide synthases (PKS), namely *ppsA-E* and *pks15/1* which constitute the PDIM + PGL locus, and that are highly conserved in PDIM/PGL-producing strains. Consensus has not been achieved regarding the genetic organization of *pks15/1* locus and knowledge is lacking on its transcriptional signature. Here we explore publicly available datasets of transcriptome data (RNA-seq) from more than 100 MTBC experiments in 40 growth conditions to outline the transcriptional structure and signature of *pks15/1*, using a differential expression approach to infer the regulatory patterns involving these and related genes. We show that *pks1* expression is highly correlated with *fadD22*, *Rv2949c*, *lppX*, *fadD29* and, also, *pks6* and *pks12*, with the first three putatively integrating into a polycistronic structure. We evidence dynamic transcriptional heterogeneity within the genes involved in phenolphtiocerol and phenolic glycolipid production, most exhibiting up-regulation upon acidic pH and antibiotic exposure and down-regulation under hypoxia, dormancy, and low/high iron concentration. We finally propose a model based on transcriptome data in which σ^D^ positively regulates *pks1*, *pks15* and *fadD22*, while σ^B^ and σ^E^ factors exert negative regulation at an upper level.

## 1 Introduction

Tuberculosis (TB) is an infectious disease caused by *Mycobacterium tuberculosis* (*Mtb)* that remains a major public health concern. In 2016, approximately 6.3 million new cases of TB were reported [1]. The mycobacterial cell wall, wherein *Mycobacterium*-specific components are located, is a crucial interface of *Mtb* and other pathogenic mycobacteria with the host [2]. Recently, Chiaradia and coworkers (2017) proposed a cell wall structure composed of three layers, namely the mycomembrane, arabinogalactan and peptidoglycan. This model proposes that the inner leaflet of the mycomembrane is composed of mycolic acids that are esterified to arabinogalactan, which in turn is covalently attached to peptidoglycan [3]. Amongst the *Mycobacterium-*specific components are two related families of glycosylated lipids: diphthioceranates (DIP) and phthiocerol dimycocerosate (PDIM), along with its variant phenolic glycolipids (PGL) [2]. PGL are known to be associated with several cellular functions, namely impermeability of the cell wall, phagocytosis [4–6], defence mechanisms against nitrosative and oxidative stress [7] and to the ability of mycobacteria to form biofilms [8, 9].

In this work, we focus on the transcriptional signature of genes comprising the biosynthetic pathway responsible for the synthesis of the phenolphthiocerol moiety of PGL, by investigating the expression of these genes when *Mtb* is grown under multiple stressors mimicking the host environment, namely pH variation, different carbon sources, limiting or excessive iron concentration, hypoxia, dormancy, phosphate depletion and antibiotic exposure. The enzymes related to the biosynthesis of PGL belong to the class of polyketide synthases (PKS). There are three types of PKS, classified according to their structure and biosynthetic function. Type I PKS contain multiple catalytic domains and can be classified as modular or iterative. Modular type I PKS have distinct functional domains that are used only once during the formation of the product. On the other hand, iterative PKS have functional domains that intervene repetitively to produce the final polyketide. Type II PKS are composed of several enzymes, each one carrying a single and distinct catalytic domain that is used iteratively during formation of the polyketide product. Chalcone synthase-like PKS, the type III PKS, represents a more divergent group that, in contrast to types I and II PKS, do not require the involvement of acyl carrier proteins (ACP) [10]. Among the genes required for PGL production are *ppsA-E* and *pks15/1* encoding type I PKS, constituting the PDIM + PGL locus, which is known to be highly conserved in PDIM/PGL-producing strains [11]. Type I PKS modules are constituted by a minimal set of three domains, namely a ketoacyl synthase (KS) domain, an acyltransferase (AT) domain, and an acyl carrier protein (ACP) domain. This module can also contain one or more of the following domains: keto reductase (KR), dehydratase (DH) and/or enoyl reductase (ER) [12]. The *pks15* encodes a KS domain while *pks1* encodes KR, DH, ER, AT and ACP domains. It has been reported that a 7 bp deletion in some *Mtb* strains, and a 1 bp deletion in a few *Mycobacterium bovis* (*Mb*) strains, leads to a frameshift that results in the split of *pks15* and *pks1* [13]. Constant and co-workers (2002) documented that, in PGL-producers, the *pks1* and *pks15* are a single gene, *pks15/1*, while they are separate (2 ORFs) in *Mtb* PGL-deficient strains such as H37Rv or Erdman. Production of PGL’s phenolphthiocerol moiety starts with the enzyme encoded by *Rv2949c* that catalyses the formation of p-hydroxybenzoic acid (p-HBA) that will later be activated by the *fadD22* product, with p-hydroxybenzoyl-AMP ligase activity [4], and finally elongated with malonyl-CoA as extender unit by *pks15/1*, in a reaction that may comprise eight to nine elongation cycles [11]. The product of *fadD29*, a fatty acyl-AMP ligase, is then responsible for activation of p-hydroxyphenylalkanoates, later transferred onto the *ppsA* product and, finally, elongated with malonyl-CoA and mehtylmalonyl-CoA by PpsB-PpsE to yield the phenolphtiocerol moiety of PGL [14–16].

As described [17], upon the entrance of *Mtb* into its target cells, a cascade of events is triggered by the immune system that, in an immunocompetent host, leads to granuloma formation and bacterial confinement. This structure is beneficial for the host, since it confines infection to localized regions, preventing bacterial spread. Some of the stresses mycobacteria are exposed to during infection include: starvation; reactive oxygen and nitrogen intermediates; hypoxia inside granulomas; iron limitation; scarcity of inorganic phosphate (P_i_); and low pH [17]. Hence, transcriptional regulators previously described to act on gene expression under such conditions were introduced into our analyses so that a comparison between our own and previous work could be established, as well as to explore the potential regulation of our genes of interest by such regulators.

Along with stress-induced genes, *Mtb* also contains a group of 13 σ subunits responsible for transcriptional regulation, namely the essential housekeeping sigma factor (σ^A^), the stress-responsive factor (σ^B^) and 11 other sigma factors that act as environmental responsive regulators (σ^C-M^). Several studies have been performed to infer the role of each sigma factor and the condition that triggers their activation, initially by analysing expression levels and by the construction of deletion strains [18, 19]. The presence and articulation of this wide variety of sigma factors enables an adaptive transcriptional response to a large set of environmental conditions. Chauhan and co-workers (2016) performed a reconstruction of the sigma factor regulatory network that enabled a clarification of the direct and indirect connections among the 13 factors [20]. This former study defined an hierarchical organization of sigma factors in *Mtb*, as well as the usage of multiple factors in response to specific stresses. Current knowledge advocates a hierarchical organization that comprises three regulation levels: (i) top level: *sigA*, *sigB*, *sigH*, *sigM*; (ii) middle level: *sigE*, *sigF*, *sigG*, *sigJ*, *sigL*; and (iii) bottom level: *sigC*, *sigD*, *sigI*, *sigK*. To get a view on which sigma factors are recruited upon the conditions that modulate *pks15* and *pks1* transcription, we therefore also introduced this breadth of sigma factors into our comparative transcriptome analyses.

As well as host-induced stress, *Mtb* is frequently exposed to drug-induced stress via antibiotic therapy. For treatment of drug-susceptible TB, a standard combination of isoniazid, ethambutol, rifampicin, and pyrazinamide is used [21]. Since TB treatment is a long-lasting process, *Mtb’s* transcriptional profile is expected to undergo defined changes along the chemotherapeutic process. *In vitro* studies have shown that for each of the above-mentioned drugs, combined expression of a set of genes results in an antibiotic resistance phenotype in *Mtb*. Since some of these drugs act on cell surfaces, the genes responsible for the corresponding drug-resistance phenotype were also included in our analysis in order to assess co-regulation of these genes with genes involved in PGL production.

Understanding the transcriptional profiles and structure of *pks15’* and *pks1* under different stress conditions that mimic the host environment is of major importance given their role in the modulation of *Mtb* and *Mb* cell surfaces, acting as the interface with the host cell and affecting pathogenicity. As such, this work aimed to elucidate the regulatory patterns responsible for controlling *pks1* and *pks15* transcription by exploring publicly available large datasets of transcriptome data (RNA-seq). This methodological approach of transcriptome profiling takes advantage of deep-sequencing technologies to get a precise measurement of transcripts at the whole genome level. Our differential expression approach enabled us to define sets of correlated genes according to their expression profiles under different stress conditions, and also to outline the transcriptional structure of the *pks15/1* locus based on the available experimental data and *in silico* predictions.

## 2 Methods

### 2.1 *In silico* analysis of regulatory data of *pks1* and *pks15*

Regulatory information on *pks1* and *pks15* were gathered from international databases such as: Mycobrowser [22], National Center for Biotechnology Information (NCBI) [23], TB Database [24, 25] and MTB Network Portal [26] (visited from 09/2018 to 01/2019). MTB Network Portal reports information produced by the cMonkey algorithm that demonstrates that *pks1* and *pks15* belong to the same two biclusters. Biclusters are sets of co-regulated genes defined by cMonkey according to mRNA-based expression levels, *de novo* identification of transcription factor binding motifs and pre-established association pathways. Location of putative ribosomal binding sites (RBS) was inferred for *pks1* with *Prokaryotic Dynamic Programming Gene finding Algorithm* (PRODIGAL) [27]. Synteny analyses were performed for *pks1*, *pks15* and *fadD22* using SyntTax (Prokaryotic Synteny & Taxonomy Explorer)[28].

### 2.2 RNA-seq data and differential expression analyses of a selected panel of genes

For expression analyses, 105 experiments (S1 Table) from *Mtb* strains (*Mtb* H37Rv and *Mtb* CDC1551), constituting a set of 40 experimental conditions in seven datasets (Accession codes at NCBI: GSE47863, GSE67035, GSE52020, GSE83814, GSE66408, GSE104599 and GSE107831) were considered. This analysis was performed for a set of 90 genes (S2 Table), including *pks1*, *pks15*, *fadD22*, *fadD29* genes comprised in bicluster modules 0211 and 0490 from *MTB* Network Portal that represent co-regulated genes, genes encoding PKS and σ factors, and genes encoding regulatory factors for each of the experimental conditions. Regarding *M*. *bovis* BCG, 21 experiments from *M*. *bovis* BCG str. Pasteur 1173P2 constituting a set of seven experimental conditions in two datasets (accession codes at NCBI: GSE66883 and GSM3160698) were analysed (S1 Table). For this analysis, a set of 50 genes were selected, including *pks1*, *pks15*, *fadD22*, *fadD29* genes comprised in bicluster modules 0211 and 0490 from *MTB* Network Portal, that represent co-regulated genes, and genes encoding PKS and σ factors. For each experiment, reads were extracted in FASTQ format using NCBI SRA Toolkit v.2.8.1.3 [29]. Those FASTQ files were mapped against a reference genome, *Mtb* H37Rv (RefSeq code: NC_000962.3, version 3) with TopHat v.2.1.0.54, [30, 31], using default settings to produce a BAM file containing a list of read alignments. Transcript identification and counting was later performed with *bias* correction by Cufflinks v.2.2.1.0 [30, 32] using as reference the annotation of the genomes listed above. Cufflinks was used to calculate the relative abundance of each gene in *Reads Per Kilobase per Million mapped reads* (RPKM). The RPKM values were transformed by log_10_ and values were normalized in relation to the housekeeping gene *sigA* expression level, heatmaps were plotted by GraphPad Prism 8 [33] and dendrograms were computed using NTSYS v2.2, calculating Pearson correlation coefficient and the *unweighted pair group method with arithmetic means* (UPGMA) as the agglomerative clustering algorithm. Pearson correlation coefficient was calculated using GraphPad Prism 8 and correlation network was plotted using Cytoscape v.3.7.2 [34, 35]. Node size of correlation networks correspond to calculated betweenness-centrality [36]. For differential expression analysis, htseq-count v.0.9.1[37] was used to count reads mapped to each gene and DESeq v.2.11.40.1 [38] was used to determine differentially expressed genes from count tables using Wald statistic test with *p*-value adjusted for multiple testing with the Benjamini-Hochberg procedure (α = 0.05). For evaluation of significance it was considered the following scale: significant (*p*-value = 0.01 to 0.05); very significant (*p*-value = 0.001 to 0.01); extremely significant (*p*-value = 0.0001 to 0.001); extremely significant (*p*-value< 0.0001). Data was plotted as heatmap using GraphPad Prism 8. The public server at usegalaxy.org [39] was used to analyse the data with NCBI SRA Toolkit, TopHat, Cufflinks, htseq-count and DESeq2.

## 3 Results and discussion

### 3.1 Revisiting the organization of *pks1* and *pks15* genetic locus across *Mtb* genomes based on predicted regulatory data and homology searches

**Fig 1:**
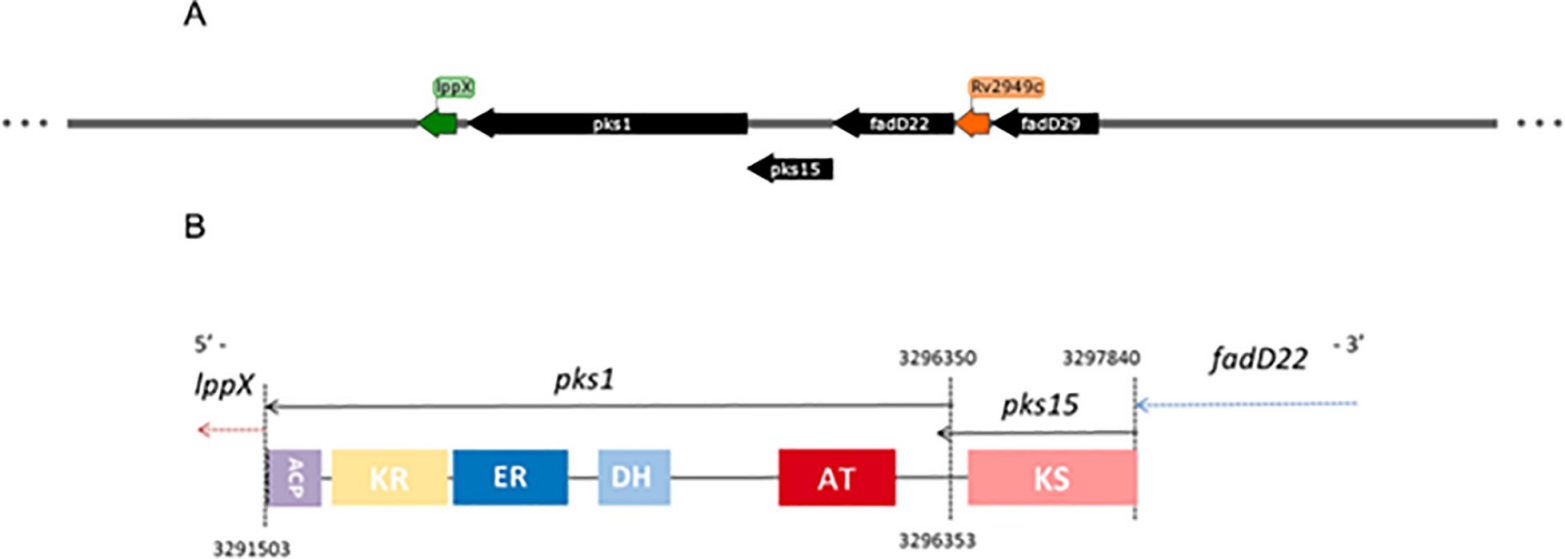
Genomic locus of *pks1* and *pks15*, protein domains and their role in the biosynthetic pathway of PGL (as described in Mycobrowser [22]). A–Schematic representation of the location of *pks1* and *pks15* in the minus strand of *M*. *tuberculosis* H37Rv genome. In black: lipid metabolism. In green: cell wall and cell processes. In orange: intermediary metabolism and respiration. B–Domain organization of Pks1 and Pks15. Abbreviations: KS, ketoacylsynthase; AT, acyltransferase; DH, dehydratase; ER, enoylreductase; KR, ketoreductase; ACP, acylcarrier protein.

Encoded on the minus strand, from position 3291503 to 3296353 for *pks1* and from position 3296350 to 3297840 for *pks15*, on the *Mtb* H37Rv genome, the *pks1* and *pks15* genes together encode a polyketide synthase with six identified domains involved in the synthesis of PGL. They appear to have a functional cooperation with *fadD22*, a bidomain initiation module. In the MTB Network Portal (retrieved on January, 2019), the *pks1* and *pks15* genes are placed together in bicluster modules 0211 and 0490, with residual values of 0.5 and 0.57, respectively, meaning that bicluster module 0211 presents a tight expression profile amongst its members, which indicates better bicluster quality and thus more certainty associated with co-expression. Furthermore, for the two genes upstream of *pks15*, *fadD22* is included in the same two modules, while *Rv2949c* is included in module 0490 (Fig 1). The mRNA-based expression levels, *de novo* identification of transcription factor binding motifs and pre-established association pathways used by the Infelerator algorithm, according to data available at MTB Network Portal, all support that *pks1* and *pks15* may be regulated by the products of seven genes: positively by *Rv0042c*, *sigK*, *Rv2258c* and *Rv3557c*; negatively by *sigB*, *Rv2745c* and *Rv3583c*. Furthermore, according to ChIP-seq data, *pks1* is bound by the transcription factor *Rv3830c* with no differential expression reported. The operon structure is undetermined; the TB Database suggests four different combinations of six genes (*fadD29*, *Rv2949c*, *fadD22*, *pks15*, *pks1* and *lppX*), while the MTB Network Portal suggests an operon composed by five genes, namely *fadD29*, *Rv2949c*, *fadD22*, *pks15* and *pks1*. All these genes are involved in the biosynthesis of the phenolphthiocerol moiety of PGL, except *lppX* that was shown to be involved in the translocation of PDIM to the outer membrane [40].

**Fig 2:**
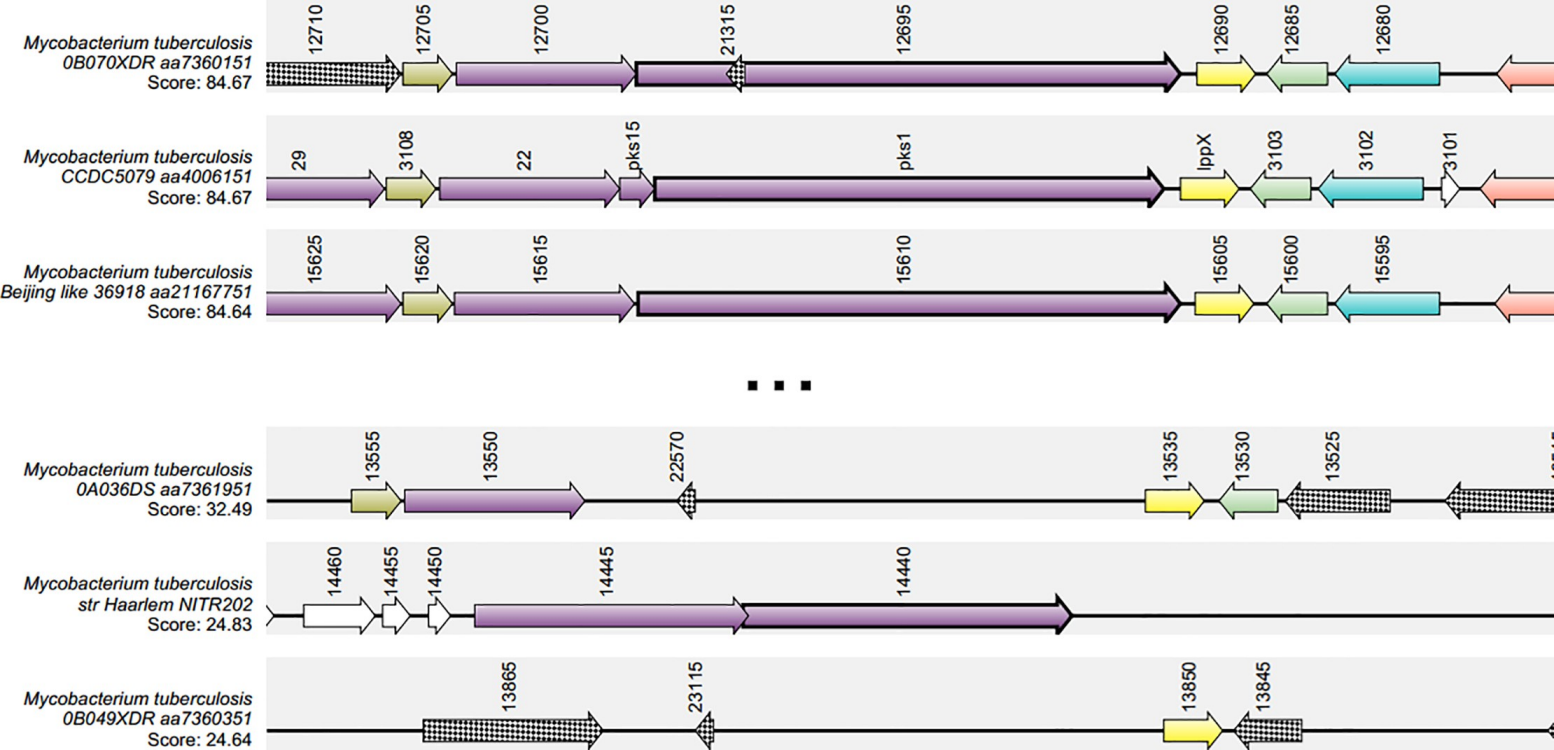
Representation of top three and bottom three scores from synteny analysis. Top 3 and bottom 3 synteny scores for *pks1* as predicted by SyntTax. The colours associated with coding sequences (CDS) facilitate the schematic representation of each CDS across the genomes analysed.

To compare the conservation of *pks15/1* locus across *Mtb* genomes, a synteny analysis was performed using the Pks1 sequence from *Mtb* H37Rv as the query. Among the 210 *Mtb* accession codes available (S3 Table), those with synteny scores above 80 were 90.5% and 99.5% for *pks15* and *fadD22*, respectively. For *pks1*, 89.5% of the accession codes used for analysis presented a score higher than 80 (Fig 2). These scores represent a normalization of the BLASTP homology score of the target protein in each genome against the reference *Mtb* H37Rv genome. When analysing local genomic conservation, an irregular pattern was noted for *pks1* and *pks15/1*, while *Rv2949c*, *fadD22*, *lppX*, *Rv2944* and *Rv2943* presented a regular organization pattern across most genomes analysed. However, when comparing the top three scoring genomes, it was also possible to identify that, for *Mtb* 0B070XDR (GenBank code: CP008970.1), the element upstream of Rv2949c did not present homology with FadD29, whilst homology was found for a protein encoded on the opposite strand of *pks1*. Many parameters influence synteny analyses, such as the assembly quality of each genome which, in the case of inadequate accuracy, may introduce mismatches and thus not reflect true polymorphisms; this may greatly impact the final output of such analyses [41]. Despite this constraint, our data does enable the recognition of a synteny block constituted by the set of genes of interest.

### 3.2 Analysis of expression data for a selected panel of genes enclosing *pks1* and *pks15*

To characterize the transcriptional signature of *pks1* and of presumably correlated genes, RNA-seq based expression analyses focused on a set of 90 genes for scrutiny, including *pks1*, *pks15*, *fadD22*, *fadD29*, as well as other genes encoding polyketide synthases and σ factors. Transcriptional profiles were compared across a set of 40 experimental conditions. Data gathered were analysed by alignment against a reference genome [*Mtb* H37Rv (RefSeq code: NC_000962.3, version 3)], by read counting and through the calculation of RPKM as a proxy for gene expression in each condition. RPKM is a relative value, meaning that it varies according not only to read count, but also to the total number of reads obtained for each experiment. In order to enable comparison across experiments, those values were normalized in relation to *sigA* housekeeping gene expression.

**Fig 3:**
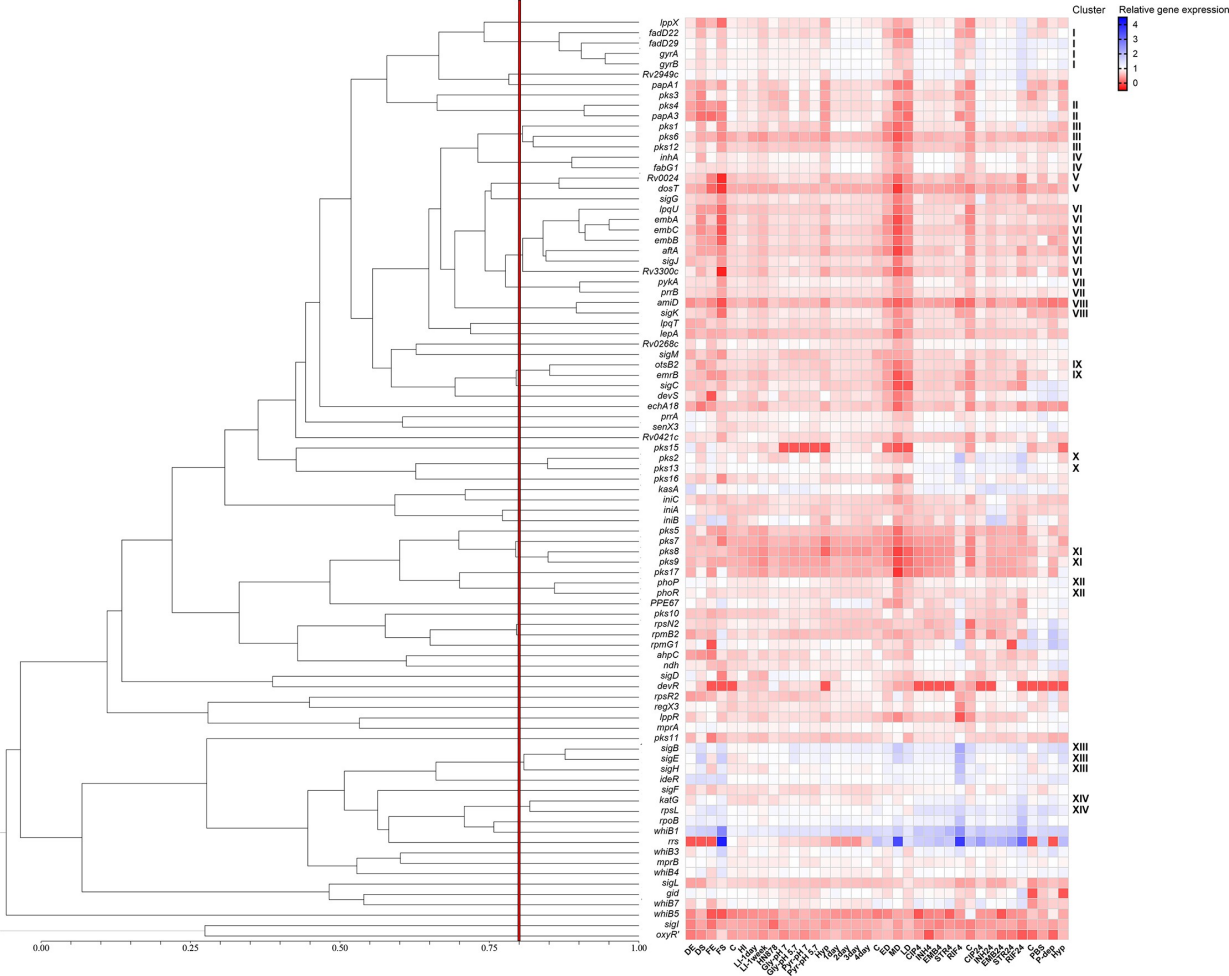
Expression profiling of selected genes from *Mycobacterium tuberculosis*, presented as log_10_ RPKM. Cut-off: 85% of similarity. Abbreviations: C—*Mtb* H37Rv grown in control conditions; DE—*Mtb* H37Rv grown in dextrose at exponential phase; DS–*Mtb* H37Rv grown in dextrose at stationary phase; FE—*Mtb* H37Rv grown in long fatty acids at exponential phase; FS—*Mtb* H37Rv grown in long fatty acids at stationary phase; HI—*Mtb* H37Rv grown in high iron concentration; LI-1day—*Mtb* H37Rv grown in low iron concentration for 1 day; LI-1week—*Mtb* H37Rv grown in low iron concentration for 1 week; HN878—*Mtb* HN878; Gly-pH 7—*Mtb* CDC1551 grown in glycerol at pH 7; Gly-pH 5.7—*Mtb* CDC1551 grown in glycerol at pH 5.7; Pyr-pH 7—*Mtb* CDC1551 grown in pyruvate at pH 7; Pyr-pH 5.7—*Mtb* CDC1551 grown in pyruvate at pH 5.7; Hyp—*Mtb* H37Rv grown in hypoxia; (1–4) day—*Mtb* H37Rv (1–4) day(s) after reaeration; ED—*Mtb* H37Rv in early dormancy phase; MD–*Mtb* H37Rv in medium dormancy phase; LD—*Mtb* H37Rv in late dormancy phase; CIP4 –*Mtb* H37Rv grown with CIP for 4h; INH4—*Mtb* H37Rv grown with INH for 4h; EMB4—*Mtb* H37Rv grown with EMB for 4h; STR4—*Mtb* H37Rv grown with STR for 4h; RIF4—*Mtb* H37Rv grown with RIF for 4h; CIP24—*Mtb* H37Rv grown with CIP for 24h; INH24—*Mtb* H37Rv grown with INH for 24h; EMB24—*Mtb* H37Rv grown with EMB for 24h; STR24—*Mtb* H37Rv grown with STR for 24h;; RIF24—*Mtb* H37Rv grown with RIF for 24h; PBS—*Mtb* H37Rv grown with PBS; and P-dep—*Mtb* H37Rv grown in phosphate depletion.

**Fig 4:**
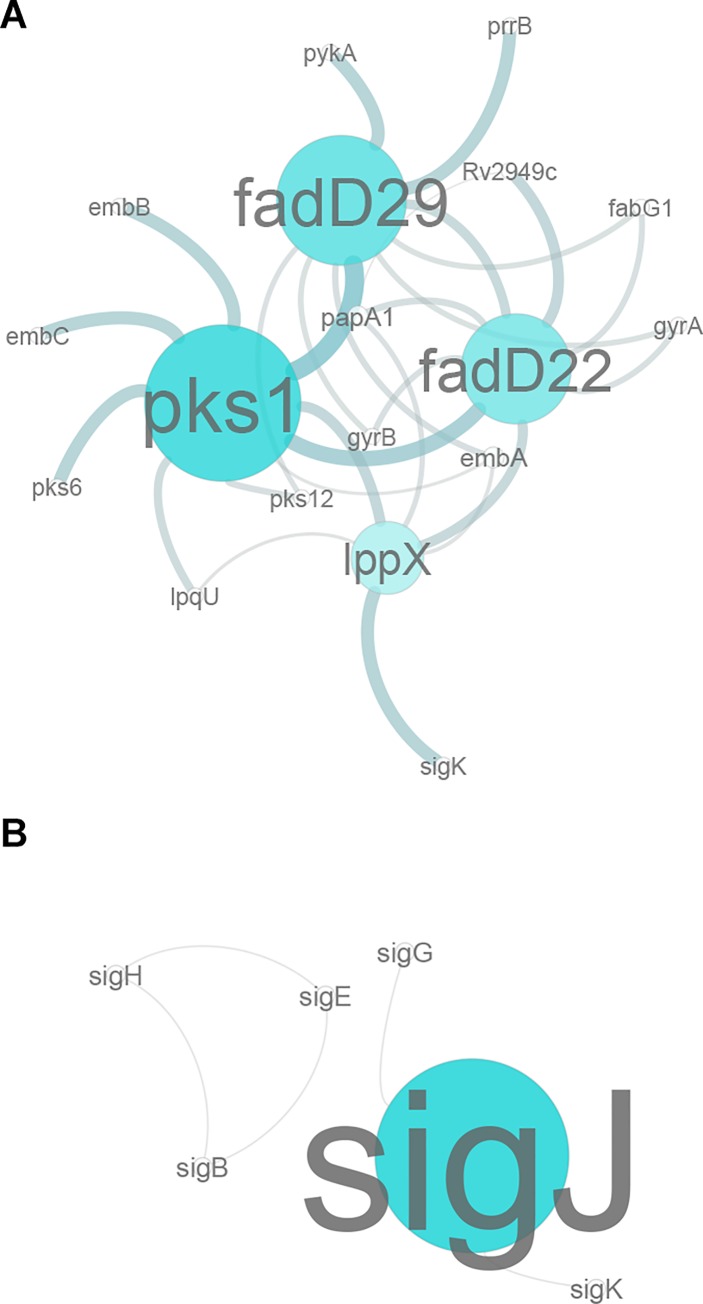
Correlation network of expression data. A: Correlation threshold = 0.75. B: Correlation threshold = 0.7. Thicker connections represent stronger correlations. Node size represents centrality values.

Besides the presence of an hypothetical synteny block across *Mtb* strains, that we herein use as our main source of data, it is known that the *Mtb* H37Rv reference strain, as well as *Mtb* CDC1551, which are core to our dataset, both present a frameshift mutation in *pks15/1*, in comparison to *Mb* BCG Tokyo which retains an intact CDS and is capable of PGL production. To understand the similarity across profiles, a dendrogram was generated, using 80% similarity as a cut-off for cluster formation; this cut-off was established to include the top quarter of similarity values in the analysis. From this, we obtained a total of 53 clusters, 39 being single member clusters. Cluster I included some of our genes of interest (*fadD22* and *fadD29*). Cluster II includes *pks1* and *pks6*, which is a *pks1* paralog, and *pks12*. (Fig 3). Another relevant cluster (II) includes *papA3* and *pks4*, both genes known to be upregulated at low pH (Fig 3). The remaining genes of interest were single member clusters. To obtain a more focused approach on direct interactions between genes, we constructed a correlation network with pairs of genes exhibiting correlation factors above 0.75. In this network, it becomes evident that *pks1* is highly correlated with *lppX* (Pearson correlation coefficient of 0.777), *fadD22* (0.811), *fadD29* (0.757) and also with *pks6* (0.807) and *pks12* (0.803) (Fig 4A).

A correlation across σ factors could also be confirmed: *sigB* is correlated with *sigE* (0.876) and *sigH* (0.833); *sigE* with *sigH* (0.783); *sigG* with *sigJ* (0.782); and *sigJ* with *sigK* (0.712) (Fig 4B). Both analyses provide evidence that *pks1* expression is highly correlated with the expression profiles of *fadD22* and *fadD29*, in agreement with reports from microarray data [42]. The expression pattern of *pks15* revealed by this analysis is strikingly different from the one found in *pks1* in some of the stress conditions under examination, turning *pks15* into a single member cluster and, consequently, absent from the correlation network (Fig 4A). In contrast with these results, available microarray data [49] suggests that *pks15* is also highly correlated with *pks1* and *fadD22*. Our analyses also suggest that *pks4* is correlated with *pks3*, as their expression profiles share 71% similarity (Fig 3), thus agreeing with previously published data [43] reporting that an *Mtb* H37Rv double mutant for *pks4* and *pks3* is not able to produce mycolipanoic, mycolipenic, and mycolipodienoic acids. Also, the fact that *pks3* and *pks4* form a polyketide structure similar to *pks15* and *pks1*, respectively, wherein *pks3* and *pks15* both encode the ketoacyl synthase domain and *pks4* and *pks1* both encode the remaining polyketide synthase domains, would suggest that *pks15* and *pks1* could also be highly correlated. Naturally, we were expecting to confirm this correlation across the selected experimental datasets.

Even though gene expression does not necessarily represent the activity of a specific σ factor, we integrated our correlation network with sigma factor expression data to plot a representation of the putative regulation of selected genes by σ factors. Six of the 13 sigma genes under analysis are highly correlated. The *sigA* factor is known to regulate *sigG*, mostly induced during macrophage infection, which will thus regulate *sigJ*, known to be overexpressed in late stationary phase of dormant cultures. *sigG* and *sigJ* will further regulate *sigL*, known to be involved in PDIM biosynthesis, that in turn regulates *sigK*, whose precise functioning remains unclear [19]. Single correlations exist between discrete sigma factors and the selected panel of genes. The *sigK* factor, which is predicted by *in silico* analysis to positively regulate *pks1* and *pks15* [44], here shows a correlation with *pks1* of 0.684 (Fig 4A). On the contrary, amongst the analyses focused on sigma factors, *sigE* was the factor that presented the lowest correlations with the established genes of interest (*lppX*, -0.567; *pks1*, -0.506; *fadD22*, -0.533; *Rv2949c*, -0.166; and *fadD29*, -0.521).

**Fig 5:**
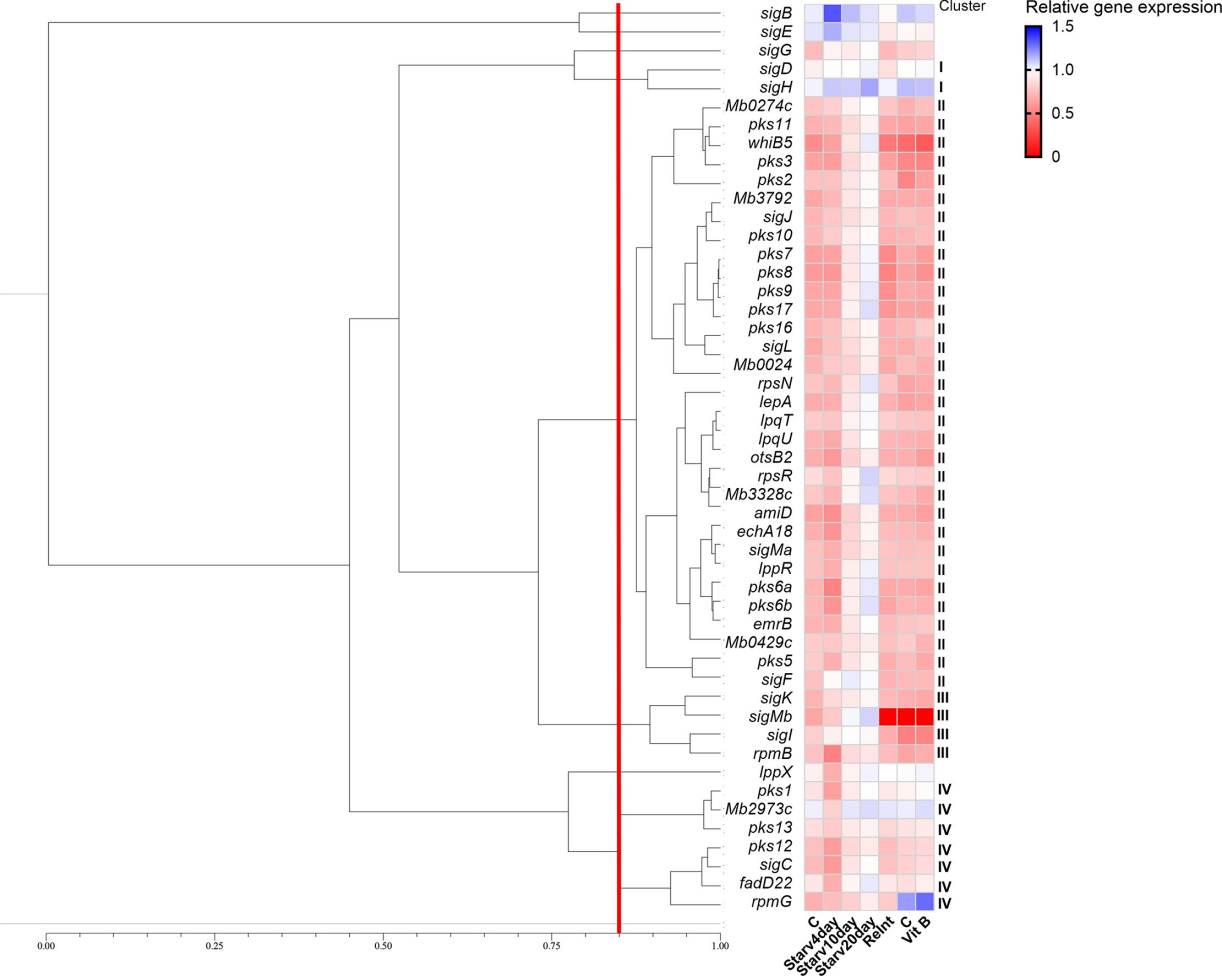
Expression profiling of selected genes from *Mycobacterium bovis*, presented as log_10_ RPKM. **Cut-off: 80% of similarity.** Abbreviations: SRR1915476/7/8 –*Mb* grown in control conditions; SRR1915479/80/81 –*Mb* grown under starvation for 4 days; SRR1915482/3/4 –*Mb* grown under starvation for 10 days; SRR1915485/6/7 –*Mb* grown under starvation for 20 days; SRR1915488/89/90 –*Mb* after reintroduction of nutrients; SRR7221299/300/301 –*Mb* grown in control conditions; and SRR7221302/3/4 –*Mb* grown with addition of Vitamin B.

For *M*. *bovis* BCG, we obtained a total of 8 clusters, with four being single member clusters using 85% similarity as a cut-off. Amongst these, it was possible to identify a cluster comprising *Mb2973c*, *pks13*, *fadD22*, *pks15/1*, *pks12*, *rpmG* and *sigC* (Fig 5). In *M*. *bovis* BCG, as verified for *M*. *tuberculosis*, *pks15/1* and *fadD22* exhibit a correlation value of 0.913, with correlation values between *pks15/1* and *fadD22* and *lppX* and *Mb2973c* all above 0.9. In addition, *lppX*, *pks15/1*, *fadD22* and *Mb2973c* were also shown to be correlated with *sigC* (above 0.79).

Comparison of normalized expression levels across *Mtb* H37Rv and *Mb* for the set of selected genes gave a correlation of 0.78, suggesting that using *Mtb* H37Rv as a reference to infer the *pks15/1* transcriptional profile across *Mtb* more broadly is a viable approach.

Expression analyses also enabled us to distinguish the expression signature of *Mtb* H37Rv from *Mtb* CDC1551 under control conditions, since the correlation factor between such strains was 0.2. This circumstance possibly reflects the impact of individual genomic differences on the respective transcriptional signature, implying that from this point onward results relative to each of these strains should be treated individually. Nevertheless, when specifically comparing *pks15* and *pks1* CDSs between *Mtb* H37Rv and *Mtb* CDC1551, they show 99.93% sequence similarity, with only a single nucleotide polymorphism in *pks15*. Even so, further genomic differences may affect overall expression.

### 3.3 Differential expression analyses

As mentioned, pathogenic mycobacteria of the MTC are subjected to a set of different growth conditions while in the granuloma and also while exposed to antimicrobial therapy. In this context, we were able to validate our analyses, as well as to perform a comparison of the differential expression of the selected genes with regulatory genes, using previously reported expression analyses. Hence in our analysis we calculated the log_2_ fold change differential expression of 90 genes, encompassing our genes of interest plus 55 genes linked to regulatory networks in each experimental condition, employing the Wald statistical test and *p*-value adjusted for multiple testing by the Benjamini-Hochberg procedure (α = 0.05),.

When comparing regular growth conditions of *Mtb* CDC1551 with nutrient-depletion and phosphate-depletion [45], the selected set of genes did not present any significant fold-changes in expression. As for the regulatory genes, significant fold-changes were only found for *sigB* in phosphate-depletion conditions.

**Fig 6:**
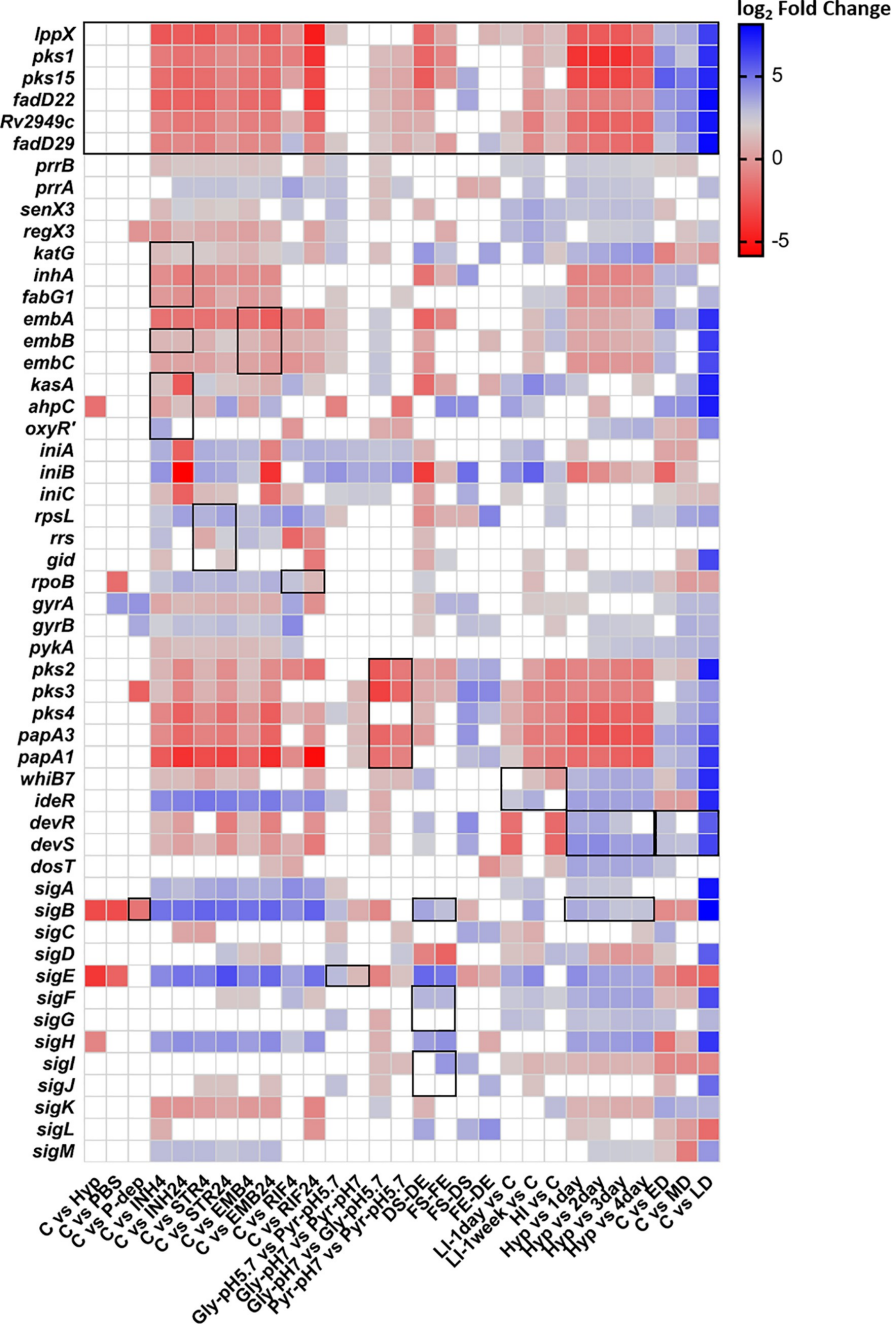
Differential gene expression represented in log_2_ fold change. Fold changes on the expression levels of genes previously described to be associated with the specific growth condition, along with our genes of interest, are identified by black outline. Blank squares represent non-significant fold-changes. Abbreviations: C–Control condition; PBS–*Mtb* H37Rv grown with PBS; P-dep–*Mtb* H37Rv grown in phosphate depletion; INH4—*Mtb* H37Rv grown with INH for 4h; INH24—*Mtb* H37Rv grown with INH for 24h; STR4—*Mtb* H37Rv grown with STR for 4h; STR24—*Mtb* H37Rv grown with STR for 24h; EMB4—*Mtb* H37Rv grown with EMB for 4h; EMB24—*Mtb* H37Rv grown with EMB for 24h; RIF4—*Mtb* H37Rv grown with RIF for 4h; RIF24—*Mtb* H37Rv grown with RIF for 24h; Gly-pH 7—*Mtb* CDC1551 grown in glycerol at pH 7; Gly-pH 5.7—*Mtb* CDC1551 grown in glycerol at pH 5.7; Pyr-pH 7—*Mtb* CDC1551 grown in pyruvate at pH 7; Pyr-pH 5.7—*Mtb* CDC1551 grown in pyruvate at pH 5.7; HI—*Mtb* H37Rv—grown in high iron concentration; LI-1day–*Mtb* H37Rv grown in low iron concentration for 1 day; LI-1week- *Mtb* H37Rv grown in low iron concentration for 1 week; Hyp—*Mtb* H37Rv grown in hypoxia; (1–4) day—*Mtb* H37Rv (1–4) day(s) after reaeration; FS—*Mtb* H37Rv grown in long fatty acids at stationary phase; FE—*Mtb* H37Rv grown in long fatty acids at exponential phase; DS–*Mtb* H37Rv grown in dextrose at stationary phase; DE—*Mtb* H37Rv grown in dextrose at exponential phase; ED—*Mtb* H37Rv in early dormancy phase; MD–*Mtb* H37Rv in medium dormancy phase; LD—*Mtb* H37Rv in late dormancy phase.

With both glycerol and pyruvate as carbon sources for growth of *Mtb* CDC1551, *pks1*, *pks15*, *fadD22*, *Rv2949c* and *fadD29* were significantly down-regulated *in vitro* at pH 7, in contrast with the *in vivo* mimicking condition at pH 5.7. In the culture grown in pyruvate, log_2_ fold change values were found to be higher than in the sample grown in glycerol. When comparing carbon sources, there is no significant difference in expression of the selected genes of interest at pH 7. However, at pH 5.7, a significant difference in *lppX* and *fadD29* expression was seen, meaning that those genes are slightly downregulated in conditions where glycerol is the sole carbon source (Fig 6). As mentioned above, the conditions explored here allow comparison between basal *in vitro* growth and *in vivo* growth inside phagosomes, where the pH is lower. It is known that the complex structure of the mycobacterial cell wall represents a major barrier to the entry of external protons [46]. Also, it is known that many acid-sensitive *Mtb* mutants present defects in genes involved in cell wall functions, and that several cell wall and lipid biosynthesis genes are differentially regulated by exposure to low pH [47]. Indeed, several of the regulatory genes reportedly responsive to acidic pH [48] were also found to be induced, namely *pks2*, *pks3*, *pks4*, *papA1* and *papA3* (Fig 6).

The comparisons between growth stages and carbon sources [49] indicated that, for both glycerol and pyruvate, *lppX*, *pks1*, *pks15* and *fadD29*, are down-regulated in the stationary phase, when compared with the exponential phase. In the cells grown in long chain fatty acids, only *pks1* and *fadD29* display extremely significant down-regulation in the stationary phase, while *lppX* and *pks15* also present significant fold changes (*p*-values are shown in S4 Table). By contrast, when bacteria were grown in dextrose, the complete set of our genes of interest was extremely significantly down-regulated in stationary phase, except for *Rv2949c* and *fadD29* that presented lower levels of significance for down-regulation (Fig 6). Since we focused on genes that are part of the biosynthetic pathway of PGL, the significant down-regulation observed when the cultures entered stationary phase may be explained by the fact that synthesis of cell wall components is reduced at this time point. Comparing dextrose, the standard carbon source used for *in vitro* growth, with long chain fatty acids mimicking the triacylglycerols available in human cells [49], we could only identify significant up-regulation of *fadD29* during the exponential phase, and of *pks15* and *fadD22* in the stationary phase, for cell growth in long chain fatty acids.

In the iron exposure assays, it was possible to observe that, after 1 day of growth under low iron concentration, only *lppX*, *Rv2949c* and *fadD29* were significantly differentially expressed, while after 1 week of exposure, the six genes were extremely significantly down-regulated when compared to the culture grown in 0.4% glucose alone. These results are also supported by the direct comparison between the two cultures exposed to low iron concentration for different periods of time, which show extremely significant up-regulation in the culture exposed for 1 day. Similar to the results from low iron concentration, exposure to high iron concentration also showed that *lppX*, *fadD22*, *Rv2949c* and *fadD29* were extremely significantly down-regulated and *pks1* was significantly down-regulated (Fig 6). We noted that our selected set of genes was highly down-regulated under low iron concentrations, which could be related with the fact that iron takes part in several biological processes inside the cell, being required for cytochromes and other hemoproteins involved in oxygen metabolism. That means that iron deprivation can affect essential cellular processes, inducing a non-replicating state, thus reducing synthesis of cell wall components [50]. Although these very interesting results were found, our data analysis does not support the currently described regulation of *ideR* and *whiB7*. Significant differential expression for *ideR* in high iron conditions was not detected, which is in conflict with reports describing its up-regulation under these conditions (Fig 6) [51, 52]. On the other hand, for *whiB7* in low iron conditions, a strong up-regulation after 6 h of exposure has been previously described, a condition for which we do not have comparable data [53, 54]. However, after one week of exposure to low iron, we found that *whiB7* was significantly down-regulated (Fig 6).

Comparing results obtained when cells were grown in hypoxia with the first 4 days after reaeration [55], *lppX*, *pks1*, *pks15*, *fadD22*, *Rv2949c* and *fadD29* were found to be extremely significantly down-regulated in hypoxia, with some of the highest log_2_ fold change values seen across all assays. Also, *lppX* and *pks1* were found to be down-regulated with extremely significant differences from the first to the third and fourth days (Fig 6). Hypoxia induces many changes in mycobacteria. Both in microaerophilic and anaerobic cultures, *Mtb* is known to develop a thickened cell wall which may be important for adaptation to low oxygen conditions [56]. However, our selected set of genes was found to be extremely down-regulated under hypoxia, agreeing with previously published data [56], and suggesting that maybe the reported cell wall thickening does involve PGL production. On the contrary, some regulatory genes, such as members of the DosT regulon (namely *devR*, *devS* and *dosT*) were shown to be highly up-regulated in hypoxic conditions (Fig 6), which is also supported by previous studies [56–58].

In the publicly available experimental data used in our analyses, dormancy was induced by growing *Mtb* in K^+^-deficient medium and, after 14–15 days of culture, adding rifampicin (5 μg/ml) to eliminate dividing bacteria [59]. By comparing cells grown to three different states of dormancy with a culture grown to log phase in standard *in vitro* growth conditions, we obtained the highest fold changes across all assays. That comparison showed extremely significant down-regulation for all genes from our defined set in dormancy conditions. When comparing between states of dormancy, it was possible to see that *pks1* and *pks15* were extremely significantly down-regulated in early dormancy when compared with mid-stage dormancy. Also, for all genes surveyed, extremely significant fold changes were found between medium and late dormancy (Fig 6). While in dormancy, mycobacteria enter a state of low metabolic activity with alteration of gene regulation in order to accumulate triacylglycerols, loss of acid-fastness and a slower growth rate. These observations explain why our selected genes of interest showed a strong down-regulation under dormancy. In agreement with previous reports, and similarly to what happens under hypoxic conditions, *devR*, *devS* and *dosT* (members of the DosT regulon) were shown to be highly up-regulated during dormancy conditions (Fig 6) [56, 57, 60].

We analysed data from drug-induced stress assays that were performed by growing *Mtb* under exposure to 0.5 μg/ml of INH, 0.5 μg/ml of STR, 1 μg/ml of EMB, and 0.25 μg/ml of RIF, separately [61]. For each drug, two time-points, 4h and 24h, were compared with reference to the control (no drug). Under exposure to INH, *lppX*, *pks1*, *pks15*, *fadD22*, *Rv2949c* and *fadD29* were significantly up-regulated at both 4h and 24h. Concerning target genes for INH exposure, *inhA*, *fabG1*, *kasA* and *ahpC* were up-regulated at both 4h and 24h; on the contrary, *oxyR’* was found to be significantly down-regulated only at 4h of exposure. For *katG* and *embB*, the data showed significant but slight up-regulation (Fig 6). Concerning STR exposure, all genes under analysis were found to be up-regulated at both 4h and 24h (*lppX*, *pks1*, *pks15*, *fadD22*, *Rv2949c* and *fadD29*), by comparison with *rpsL*, *rrs* and *gid*, which showed significant differential expression but with small fold changes (Fig 6). When cells were exposed to EMB, *lppX*, *pks1*, *pks15*, *fadD22*, *Rv2949c* and *fadD29* were significantly up-regulated at both 4h and 24h. The genes *embA*, *embB* and *embC* were also significantly up-regulated in the cultures exposed to EMB, which agrees with previous published data and validates our findings (Fig 6) [61]. The exposure to RIF leads to the up-regulation of the selected panel of genes, with significant fold changes at both 4h and 24h, with more pronounced fold changes after 24h of exposure (Fig 6).

**Fig 7:**
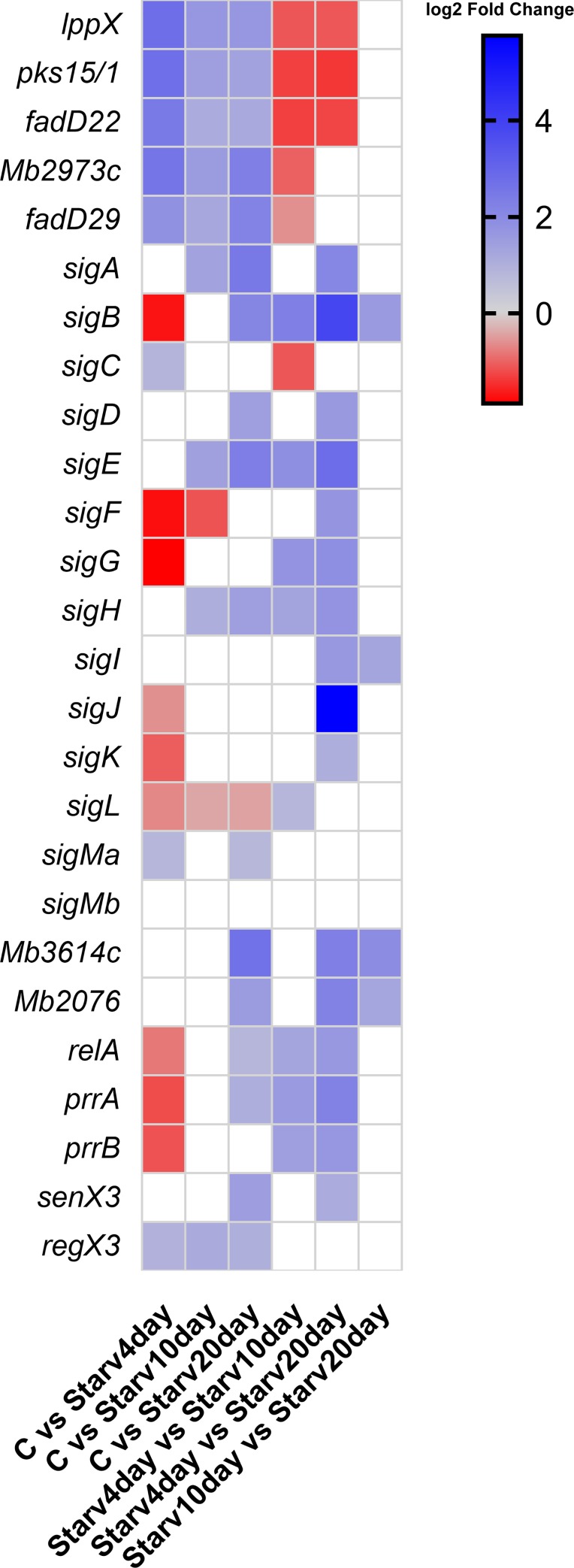
Differential gene expression represented in log_2_ fold change. Blank squares represent non-significant fold-changes. C–Control condition; Starv4day–*Mb* grown under starvation for 4 days; Starv10day–*Mb* grown under starvation for 10 days; and Starv20day–*Mb* grown under starvation for 20 days.

For *M*. *bovis* BCG, data from starvation assays were collected after 4, 10 and 20 days and after reintroduction of nutrients [62]. For the first three conditions, all genes of interest were up-regulated [*lppX* (*p*-value = 1.5462x10-42; *p*-value = 1.5347x10-17; *p*-value = 3.4984x10-18), *pks15/1* (*p*-value = 3.2164x10-30; *p*-value = 1.3595x10-8; *p*-value = 5.5910x10-8), *Mb2973c* (*p*-value = 3.7632x10-12; *p*-value = 8.8716x10-5; *p*-value = 6.1298x10-10) and *fadD29* (*p*-value = 2.6748x10-5; *p*-value = 0.0107; *p*-value = 3.3421x10-7)] when compared to the control. The differential expression of genes reported to play a regulatory role under starvation conditions in *Mtb*, such as *Mb3614c* (*p*-value = 2.7774x10-5), *Mb2076* (*p*-value = 0.0034), *relA* (*p*-value = 0.0127), *prrA* (*p*-value = 0.0125), *senX3* (*p*-value = 5.9579x10-5) and *regX3* (*p*-value = 7.6639x10-12), was also evaluated, whereby after 20 days of starvation up-regulation of these genes was noted (Fig 7). In the assays involving the introduction of vitamin B [63], no differential expression was evidenced.

## 4 Concluding remarks

*Mtb* virulence is related to its ability to survive inside macrophages. During infection, macrophages engulf bacilli, constituting a hostile intracellular environment for bacterial replication. Yet *Mtb* can overcome these macrophage defences in a coordinated and complex process, allowing intracellular growth and persistence. Recent models of persistence inside the host point to bacterial subpopulations in a latent state that maintain their ability to reactivate upon host immunosuppression [17, 64, 65]. PGL is an important *Mtb* virulence factor and its production involves several PKS, such as *pks1* and *pks15*, which have been shown to have a critical role in PGL biosynthesis, since the presence of a frameshift mutation that disrupts the *pks15*/1 CDS was associated with the lack of PGL production in *Mtb* [13]. Also, it is known that the reference strain for pathogenic mycobacteria, *Mtb* H37Rv, as well as the common *Mtb* CDC1551 strain, also contain this frameshift mutation, while other *Mtb* strains circulating across the world contain an intact *pks1/15* locus. Inferring the regulatory pattern of *pks1* and *pks15*, using a genome-wide approach by analysis of RNA-seq data, could unveil the regulatory patterns controlling phenolphtiocerol and phenolglycolipid production in pathogenic mycobacteria and, indirectly, shed light on the downstream processes in which these molecules participate.

The analysis of expression data gathered from publicly available sources suggested that the target genes selected for this work, *pks1* and *pks15*, may be transcribed as a polycistronic unit composed by three to six genes located both upstream of *pks15* and downstream of *pks1*. All these gene products, except FadD29’, take part in the biosynthetic pathway of the phenolphtiocerol moiety of PGL. Also, *pks1* and *pks15* both seem to be positively regulated by *sigK* and negatively regulated by *sigE*, based on algorithmic predictions [26].

By clustering the expression data from more than 100 RNA-seq datasets for *Mtb*, in a robust set of 40 growth conditions, it was possible to correlate *pks1* expression with that of *fadD22* and *pks6*. With a closer analysis, focused on the correlation coefficient values, we were able to confirm that all genes thought to belong to the putative polycistronic structure present similar expression profiles. Correlations between these genes were shown to be above 0.70, except for *pks15*. Also, we found that the *pks1* correlation coefficient values were above 0.80 with *pks6* and *pks12*. As noted, *pks15* did not show such high correlation values, although this is mostly due to the presence of several null RPKM values and not to the dissimilarity of the expression profile, since the reads mapped to *pks1* in strains without the *pks1/15* frameshift (e.g. BCG). In this integrative analysis, it was also possible to link genes encoding σ factors with the selected genes of interest; e.g. we found a correlation coefficient of 0.8 between *sigK* and *lppX*. While these results must be treated with caution, since σ gene expression may not reflect effective factor activity, they offer new insights into the potential function of sigma factors whose functional role remains unclear, such as SigK.

As referred to previously, mycobacteria are subjected to several stress conditions while inside macrophages. By analysing the differential expression of *lppX*, *pks1*, *pks15*, *fadD22*, *Rv2949c* and *fadD29*, it was possible to define under which conditions these genes are positively or negatively regulated. We analysed expression levels of strains grown under a diverse set of conditions, namely pH, carbon source, hypoxia and phosphate depletion for *Mtb* CDC1551, growth phase, exposure to limiting or excessive iron concentration, hypoxia, dormancy, and antibiotic exposure for *Mtb* H37Rv. This analysis revealed that our selected genes of interest are up-regulated at acidic pH (in *Mtb* CDC1551) and antibiotic exposure and down-regulated at stationary phase (in *Mtb* CDC1551), under hypoxia and dormancy, and at both low and high iron concentrations. The combination of two sets of data, i.e. clustering of genes by expression data and differential expression analysis, suggests that *fadD29* may be set apart from the other genes in the set. Also, in one of the conditions, *fadD29* expression seemed to diverge from that of *lppX*, *pks1*, *pks15* and *Rv2949c*, and in another, both *Rv2949c* and *fadD29* expression profiles diverge from *pks1* and *pks15*. Using differential expression analysis, we were also able to confirm that *sigK* shares the expression profile with the selected genes of interest in 88% of the exploited conditions with significant fold-changes; almost the same percentage is also verified for *sigJ*. On the contrary, *sigE* presents approximately 90% of expression profile dissimilarity with the selected panel of genes in the conditions under analysis, with significant fold-changes, as well as *sigB*.

While for *Mtb* we were able to gather a robust set of data from previous studies, for *M*. *bovis* BCG it was only possible to collect data from seven growth conditions, of which three represent regular *in vitro* growth, three represent growth under starvation at three time-points, and one represents the addition of vitamin B. This smaller data set led to a lower number of clusters. Interestingly, the members of the putative polycistronic structure, except for *lppX*, cluster in the same group. This analysis is in full agreement with the one performed for *Mtb* as most genes of interest are contained in the same cluster. Also, expression values of *pks1* appear to be similar in control assays performed in *Mb* and in *Mtb* H37Rv for which relative gene expression has a correlation of 0.78, indicating that although PGL production may be abolished in *Mtb* H37Rv, the *pks1* transcript is similarly expressed in both *Mtb* H37Rv and BCG, suggesting a secondary role for this transcript.

**Fig 8:**
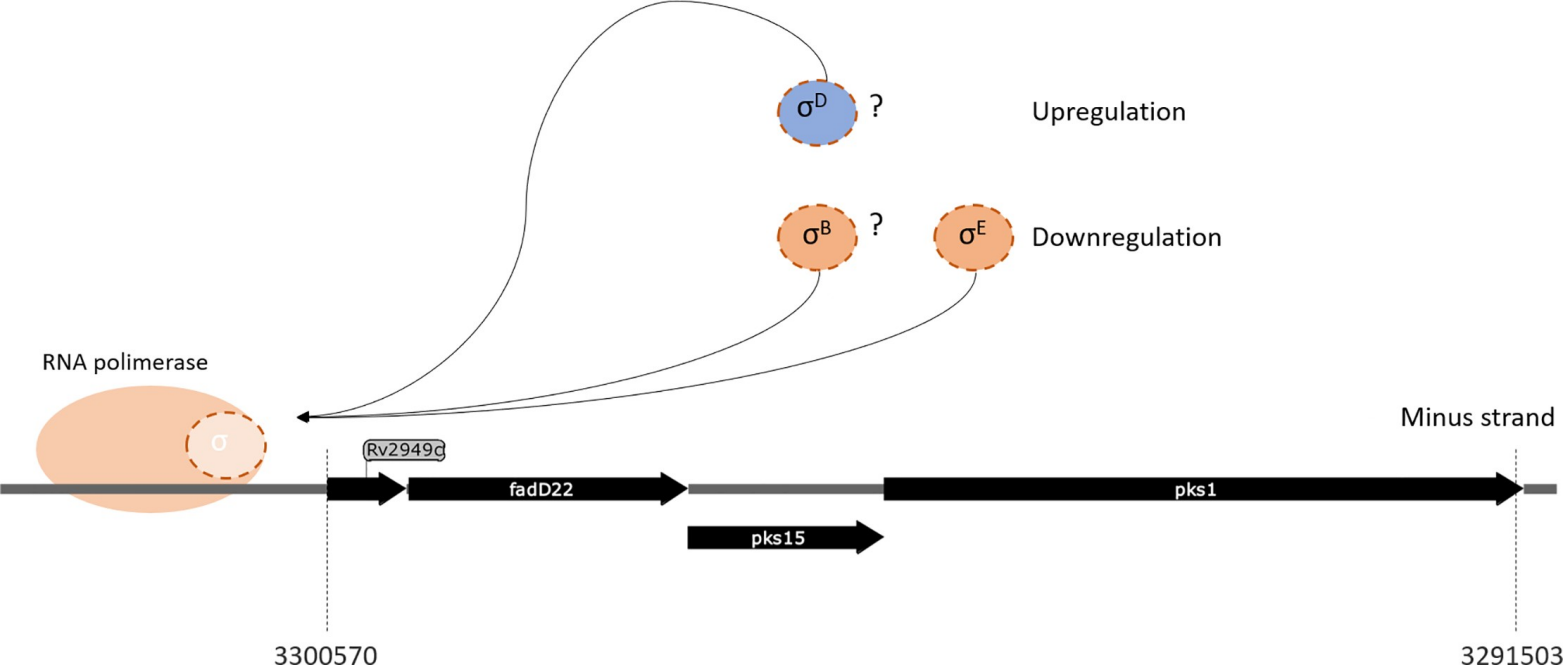
Schematic representation of the proposed polycistronic structure model. The *pks1*, *pks15* and *fadD22* genes are represented with putative regulation from σ^D^ (positive), and σ^B^ and σ^E^ (negative).

Building on the information from previously published reports and the transcriptome data we retrieved, compiled and analysed here, we propose a regulatory model for *pks1* and *pks15*. In this model, we use a conservative approach selecting genes coherently sharing expression patterns and exhibiting functional similarity when considering a polycistronic structure model. For this model, we selected a set of four genes, *pks1*, *pks15*, *fadD22* and *Rv2949c*, that fulfil the criteria stated above. Based on differential expression analysis, we also selected a set of three σ factors (σ^D^, and σ^B^ and σ^E^) that seem to be involved in the regulation of *pks1*, *pks15* and *fadD22* expression (Fig 8). Both σ^K^ and σ^E^ were previously computationally predicted to regulate the genes belonging to this polycistronic structure according to mRNA-based expression levels [66], which is coherent with the expression data analyses reported herein. However, σ^D^ and σ^B^ exhibited similar expression patterns and thus are also included in our proposed model. The genes encoding factors σ^D^ and σ^K^ were shown to be down-regulated under hypoxia and dormancy, as well as in stationary phase. On the contrary, the genes encoding σ^B^ and σ^E^ factors were up-regulated under the same conditions, with *sigB* being previously shown to be up-regulated under hypoxia [18]. While σ^D^ and σ^K^, that appear to positively regulate the selected genes of interest, belong to the lower level of σ factors regulation, the σ^B^ and σ^E^ factors, which putatively regulate in a negative way the selected genes of interest, belong to a hierarchically upper level of regulation. Also supporting the hypothesis that this putative polycistronic structure is regulated by σ^B^ and σ^D^ are some studies analysing the expression profile of knock-out mutants for those sigma factors. For σ^B^ it was reported by Lee and coworkers (2008) that genes encoding for proteins involved in cell wall processes are highly upregulated in complementation mutants, which is in contrast with our data analyses [67]. On the other hand, a *ΔsigD* mutant showed reduced expression of genes involved in the synthesis of phospholipids and fatty acids [68]. Even though our data analyses suggests that σ^K^ is a regulator of this set of genes, previous analyses of an *Mtb* *ΔsigK* mutant reported no differential expression of our genes of interest [69], leading us to exclude this sigma factor as a hypothetical regulator in our model.

Further experimental validation of our findings and proposed regulatory model could be achieved via ‘classical’ experiments such as Northern blots, construction of *pks1* and *pks15* knock-out mutants, and transcriptional fusions with reporter genes of the upstream regions of the constituent genes in this putative polycistronic unit in order to unveil the exact location and activity of the promoter. Analysis of *pks1* and *pks15* expression in mutant strains of their putative regulators under several growth conditions would further serve to validate the global networks that exert effects on *pks1* and *pks15* activities, genes that play a crucial role in PGL production and thus act at the interface of host-pathogen interaction.

## Supporting information

S1 Table (XLSX)

S2 Table (XLSX)

S3 Table (XLSX)

S4 Table (XLSX)
